# Co-Creation with Consumers for Packaging Design Validated through Implicit and Explicit Methods: Exploratory Effect of Visual and Textual Attributes

**DOI:** 10.3390/foods11091183

**Published:** 2022-04-19

**Authors:** Laura López-Mas, Anna Claret, Alejandra Bermúdez, Mar Llauger, Luis Guerrero

**Affiliations:** 1 Food Quality and Technology, Institute of Agrifood Research and Technology (IRTA), Finca Camps i Armet, s/n, 17121 Monells, Spain; laura.lopezm@irta.cat (L.L.-M.); anna.claret@irta.cat (A.C.); alejandra.bermudez@irta.cat (A.B.); mar.llauger@irta.cat (M.L.); 2 Department of Agri-Food Engineering and Biotechnology (DEAB), Universitat Politècnica de Catalunya (UPC), Baix Llobregat Campus, Building D4, st/ Esteve Terradas, 8, 08860 Castelldefels, Spain

**Keywords:** claim, informative, interpretative, new product development, implicit measurements

## Abstract

Packaging is no longer a mere structural element that only aims to preserve foods, but it is also a powerful marketing tool able to affect product perception, purchase decision and consumers’ food choices. Incorporating consumers’ voices into packaging design through co-creation could maximise its impact on the market. The main goal of this exploratory study was to test the usefulness of co-creation with consumers for packaging design. For that purpose, a survey with 200 Spanish participants was conducted to find out which of the presented visual and textual packaging attributes were the most appropriate. A validation study with 40 participants using implicit (eye tracker, galvanic skin response and automatic facial expression analysis) and explicit measurements was used to test the packaging co-created by consumers against some of its possible competitors in the market. The co-creation process with consumers allowed for the identification of the visual and textual attributes, among the available options, that best fit their preferences, whereas the validation process confirmed that the packaging design co-created by consumers was equally or even preferred over the competitors. The information gathered might help designers and marketers to guide the packaging design for fish products in the Spanish market.

## 1. Introduction

Currently, consumers have more options than ever when shopping, as they can choose between a large variety of food products that are usually in stock at a regular supermarket. Most purchasing decisions, approximately 76%, are made at the point of purchase [1]. In-store buying decisions are sometimes impulsive, especially when it comes to low-involvement products such as foods [2]. In this sense, packaging is able to grab a consumer’s attention at the point of purchase and allow for the differentiation of a product from the competition. Indeed, according to van Rompay and Veltkamp [3], food packaging could be the most direct and influential communication element at the point of purchase. Therefore, packaging is no longer a mere structural element that only aims to store and preserve foods, but it is also a powerful marketing tool able to affect product perception, purchase decision and consumers’ food choices [4,5,6].

When designing food packaging, there are many attributes to consider to ensure that it attracts consumer attention in the marketplace, transmits the most effective message and enhances consumers’ experience [7]. A main distinction can be made between packaging elements: (i) visual attributes, which draw attention, transmit non-verbal information, and mainly affect people’s emotions (for example, colour); and (ii) textual attributes (or informational), which transmit verbal or numerical information and are more likely to affect people’s cognition (for example, claims) [2,5,8]. Other packaging cues less explored may also play a relevant role in consumers’ perceptions, such as tactile and auditory attributes, as demonstrated by Labbe et al. [9] for consumers’ expectations of food naturalness.

In turn, textual cues can be divided into two main domains, namely: (i) informative claims, also known as objective or reductive, that provide impartial and objective information (for example, the exact number of calories), and (ii) interpretative, also known as evaluative, which provide information with some kind of processing to aid consumers in reducing the cognitive burden needed to interpret its meaning (for example, low in calories) [2,6,10].

Several authors have studied the role that visual attributes of packaging play in consumers’ preferences and evaluations of food products such as colour [8,11], visibility (window presence) [7], photography [7,8,12,13], typeface [14,15], presentation of the product inside the packaging [16] and quantity [8,16]. The role of textual attributes in consumers’ preferences has also received attention, for example, claims regarding convenience [11], taste [11,13], health [12,17,18], nutrient content [18,19,20], freshness [11], sustainability [21], environment [22] and processing [19]. However, despite the multiple studies on the topic, most investigations focused on the separate functions of the packaging (such as visual or textual cues) rather than analysing them in a holistic way [5]. In addition, some of those studies started from a premise that considered only a few packaging attributes, preselected by the researchers, instead of considering a larger number.

Approximately 80% of new food products launched on the market fail within the first year [23], in some cases, as a result of poor packaging design that does not entirely satisfy consumers’ demands and expectations [8]. Therefore, understanding consumers’ responses to food product packaging seems to be relevant for maximising package impact [8], and one of the best ways to do so is by incorporating consumers’ voices into new product development (NPD) through co-creation [24,25]. Co-creation, defined as collaboration between firms and consumers [26], has proven to be a useful tool for inquiring into consumers’ wants and needs during NPD [27,28]. However, despite its potential usefulness, no study has been found using co-creation with consumers in packaging design, considering visual and textual attributes, and focusing on multiple attributes at the same time.

Several implicit techniques can be useful for testing the effectiveness of involving consumers in packaging design in addition to traditional explicit techniques (for example, surveys), which often are not able to capture unconscious motives, emotions and associations of individuals [29]. Implicit techniques have the major strength of providing a range of more objective measurements [30], as some social conventions may constrain the expression of individuals’ opinions [31] gathered through explicit measurements. Various authors successfully used several implicit techniques during packaging design, including eye trackers (ETs) [30,32], galvanic skin response (GSR) [33] and automatic facial expression analysis (AFEA) [34,35,36]. ETs are capable of capturing visual attention and objectively assessing consumers’ perceptions of visual stimuli, such as food packaging [32], using eye movement as an indicator of information acquisition behaviour [37]. GSR measures the changes in the electrical conductance of the skin that occur when glands secrete sweat, which are indicative of sympathetic activity and emotional intensity [38,39]. AFEA is a means of emotional valence measurement (positive vs. negative emotions). Computer-based facial expressions analysis systems capture and map facial configurations to class them into emotion predictors using computer-vision algorithms [40].

Implicit techniques have long been debated on its ecological validity, which refers to whether or not the study findings in a laboratory setting can be generalised to real-life [41]. Ecological validity greatly depends on the techniques used, for example, it is higher for screen-based ET and lower for electroencephalography (EEG) [42]. In addition, the type of stimuli also can affect ecological validity. In other words, if standard stimuli are used in emotional research, the contextual information that makes emotions meaningful is normally lost, thus causing a decrease in its ecological validity [43]. For this reason, the combination of implicit and explicit techniques is often applied, as it provides complementary information [29], consequently, allowing one to overcome some of the main limitations associated with each technique.

Product category usually influences the packaging design [18,44]. Fish is a product that, historically, was commercialised unpackaged or with minimal packaging, as it was mainly sold unprocessed. However, in recent years, packaged fish products appeared on the market to satisfy consumers’ demands for more convenient products, more detailed information provision (labelling) and more transparent pricing [45]. Despite its increasing visibility, fish packaging has received little attention among researchers [46]. Indeed, there are only a few published studies that focus on fish and fish product packaging [11,45,47].

The main goal of this paper was to explore, through implicit and explicit techniques, the usefulness of co-creation with consumers for packaging design of fish products, thus including visual and textual attributes.

## 2. Materials and Methods

This study analyses the effectiveness of co-creating packaging with consumers. Given the large number of attributes that normally define a package (colours, shape, size, claims, etc.) and the possible interactions between them, we opted for a holistic exploratory approach despite all the limitations associated with it, which are discussed in Section 3.5. The alternative would have been to only focus on some aspects of the packaging, but in that case, we would have moved further away from the real situation in which the packaging is usually evaluated as a whole.

### 2.1. Participants

A sample of 200 participants was recruited in Spain. A convenience sampling method was applied, which included quotas for gender (evenly split) and age (between 18 and 64 years). All participants were fish consumers (once a month, at least) and responsible for food purchase and preparation within their household. A subcontracted market research agency oversaw the recruitment of the participants and the launch of the online survey.

For the validation study, 40 respondents were recruited in Spain through a convenience, intentional, and reasoned sampling. All of the participants were older than 18 years, fish consumers (once a month, at least) and responsible for food purchase and preparation within their household. In addition, they met several specific criteria to take part in the implicit experiment [40,48,49] including not wearing glasses (except for monofocal) and neither having had eye surgery nor suffering from any eye disorders or diseases (for example., strabismus, amblyopia (lazy eye), mydriasis (permanently dilated pupils), daltonism (colour blindness), cataract and glaucoma). No items that covered participants’ faces (for example, bushy beard, facial tattoos, and thick-rimmed glasses) or substances that hindered their facial mobility (e.g., Botox) were allowed.

### 2.2. Questionnaire

The co-creation process for packaging design focused on the fish product “meagre burger with mushrooms (black trumpet)” developed within the European Horizon 2020 funded project MedAID (see the Funding section for more details). The product was selected based on its convenience, but also on its novelty, as its ingredients (meagre and black trumpets) are seldom used in the Spanish market. The questionnaire was divided into three main parts: (i) visual attributes; (ii) textual attributes; (iii) sociodemographic characteristics (such as gender, age, education level, household size and presence of children at home).

The visual attributes preferred by participants were identified by means of a compositional approach [50], similar to the self-explicated measurements, which are particularly useful for measuring consumers’ preferences when many attributes are considered simultaneously, although it does not allow for an exploration of the interaction between them [51,52]. Participants were asked to select which visual attributes they preferred among different options. When dealing with low involvement products, such as food, preference is a good predictor of purchase intention [53]. The visual attributes of the packaging included the container type (bowl, bag, tray and box), colour (61 different options), window presence and type (16 options), picture presence and type (6 options), typeface (15 options), package presentation (individual, per serving and without divisions) and quantity of the package (one, two, four and more than four servings). An extract of the questionnaire used to assess preferred visual attributes for consumers can be found in Appendix A.

The textual attributes of the packaging were grouped into three dimensions of quality: (i) searched attributes, those that are available before consumption; (ii) experienced attributes, which can only be evaluated after consumption; and (iii) credential attributes, which cannot be evaluated even after normal use of the product [54,55,56,57]. Three rating-based full-profile conjoint analyses were performed, one per each quality dimension, to determine the relative importance of each factor in the consumers’ purchase intention, measured on an 11 point probability scale (0 = “absolutely no chance” to 10 = “absolute certain to buy”) [58]. Four factors were included in each conjoint analysis: (i, searched quality) convenience, price, products’ presentation and recyclability; (ii, experienced quality) freshness, texture, flavour and novelty; (iii, credential quality) health, natural, animal welfare and sustainability. Within each factor, two ways (levels) of deliver textual claims were tested: informative vs. interpretative. Two main approaches were used to select the textual attributes: a literature review and an assessment of current food products available in Spanish market. The information and attributes that were not considered were those that are mandatory on European fish labels such as production method (wild/farmed fish) or date of minimum durability [59].

### 2.3. Packaging Design

Four mock-ups of fish burger packaging were designed using Photoshop, version 21.2.1 (2020) (Adobe Inc., Mountain View, CA, USA) (Appendix B) and used as stimuli in the validation study. The mock-ups used were: (i) packaging of the preferred visual and textual attributes for the participants: the visual attributes most frequently chosen and the textual claims (levels from the conjoint analysis) with higher utility from the factor with higher relative importance for each of the quality dimensions; (ii) packaging of the second preferred visual and textual attributes chosen by the participants; (iii) packaging from an owned brand in Spanish supermarkets with the largest market share; (iv) packaging from the best-known and well-established brand of fish products in Spain. The former two packages were co-created by participants while the last two fish burgers packages from the Spanish market were regarded as possible competitors. The selection of two completely different packages for both the co-created and the competing products was made to reduce the possible bias caused by the inference of a particular attribute. In other words, to avoid that a specific attribute might triggers participants’ preference regardless of the rest of the packaging. The inclusion of multiple attributes in the co-created packages allowed the designing of a realistic packaging to be compared with real competitor products. Conversely, the two competing products were slightly modified to ensure that participants focused on the packaging rather than the product itself. To this end, the two packages from the competitors were photographed and photo edited (for example, brand name was removed and replaced with the packaging background, salmon fish burgers were replaced by meagre fish burgers with mushrooms to avoid respondents making their decisions based on the fish species, etc.). We do not have information on how the two competing packaging were created, neither if consumers took part in their design. However, this is not a critical aspect, as the goal of our study was to explore the usefulness of co-creation with consumers for packaging design, this is whether a packaging designed by means of co-creation can become a real competitor of successful products already existing in the market.

### 2.4. Validation Procedure

**Figure 1:**
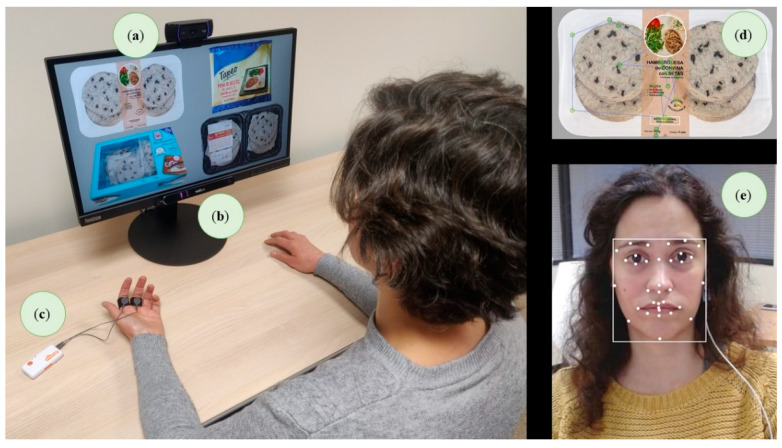
Experimental set up for implicit measurements acquisition in the validation study: (**a**) camera for automatic facial expression analysis; (**b**) screen-based eye tracker; (**c**) galvanic skin response Bluetooth sensor; (**d**) example of a gaze path collected with the eye tracker; (**e**) example of automatic facial expression analysis. Informed consent was obtained from all participants.

The validation process included a combination of implicit (ET, GSR and AFEA) and explicit measurements. The equipment used to gather implicit measurements were ET (Tobii Pro Nano, Tobii Pro, Stockholm, Sweden), GSR sensor (Shimmer3 GSR+, Shimmer Research, Dublin, Ireland) and a camera for AFEA (AFFDEX, Affectiva Inc., Boston, MA, USA) (Figure 1). Prior to the start of the test, participants received all the information needed to decide if they wanted to take part in the experiment in an informed way (informed consent). A calibration process preceded the data acquisition. To increase the ecological validity of the gathered data, a shopping setting was evoked. The participants were shown with a picture of a trolley in a supermarket and the context was evoked by telling them to imagine themselves in there doing shopping. The first task carried out by participants was to look at the four packaging mock-ups presented simultaneously for 30 s, trying to emulate a real situation in a supermarket (implicit measurement). To avoid the effect of the presentation order of the images, four balanced orders were established following the William’s Latin square [60]. Next, respondents were asked to rank their preference for each packaging from 1 to 4 as well as to punctuate the acceptability of each one on a continuous scale from 0 to 10 (0 = “lowest acceptability” to 10 = “maximum acceptability”) (explicit measurement). Following this, respondents were presented sequentially with each image individually for 10 s to allow them to observe the details of the packaging (implicit measurement). Afterwards, they were presented with the four packages simultaneously and asked about their purchase intention for each one on an 11 point probability scale (0 = “absolutely no chance” to 10 = “absolute certain to buy”) [58] (explicit measurement). Finally, more in-depth questions were formulated for each packaging to inquire into the consumers’ perceptions (for example, what were the positive and the negative aspects) through a personal interview (explicit measurement).

### 2.5. Data Analysis

Visual attributes were analysed by means of absolute frequency of selection, as they were single-choice questions. To estimate the utility values and the relative importance that participants placed on the textual attributes within each quality dimension, the “conjoint analysis” function from the XLSTAT software, version 2020.1 (2020) (Addinsoft, Paris, France) was used, which also served to perform all statistical analyses. A one-way analysis of variance (ANOVA) with Tukey’s honestly significant difference (HSD) post hoc test was used to determine if there were statistical differences (*p* < 0.050) among levels and factors of the conjoint analysis.

Implicit measurements were divided into two elements: (i) visual attention (qualitative and quantitative, measured with the ET) and (ii) emotional response (intensity and valence, measured with the GSR and the AFEA, respectively). Qualitative visual attention was derived from a heatmap that displayed the location and density of the gaze fixations. Quantitative visual attention was calculated using eye tracking metrics, although previously, each package design was defined as an area of interest (AOI), which allowed to compare metrics among them. Three eye tracking metrics were retained: (i) time to first fixation (TTFF), which indicated the amount of time (ms) that it took, on average, to look at a specific AOI for the first time; (ii) fixation count, the count of all gazes fixated for more than 100 ms inside an AOI; (iii) revisit count, the count of the re-examination of an AOI [48,61,62,63]. On the other hand, to measure emotional response, AFEA analyses were used to detect emotional valence by means of an AFFDEX classification algorithm to compare numerically the facial configurations of the respondents with normative databases [40]. The amount of time participants displayed positive or negative expressions in relation to the total presentation time of each packaging was computed in percentage. A 30% probability amplitude-based thresholding was used for individual facial response detection [40], as a moderate response was expected due to the type of stimuli [48]. Facial expressions that increased the likelihood of positive valence included a smile and raised cheeks, while those that increased the likelihood of negative valence were internal brow lift, brow furrow, nose wrinkles, upper lip lift, lip corners depressor, chin lift, lip press and lip suction [64]. To analyse emotional intensity, an automatic GSR peak detection algorithm was applied to the GSR calibrated signal to capture variations in its phasic component [65]. For each participant, the total number of peaks detected in relation to the total presentation time of each packaging, expressed as peaks/min, was calculated. To capture, analyse and integrate implicit measurements, the iMotions Research Software (iMotions A/S, Denmark) was used. To determine if there were significant differences among the four packaging (AOIs) eye tracker metrics, an ANOVA with Tukey’s test was used for TTFF, whereas a k proportion test after a pairwise comparison with the Marascuilo procedure was used for fixation and revisit count. An additional ANOVA with Tukey’s test was used to determine if there were significant differences in AFEA and GSR measurements among the four packages, that is, if there were differences in the amount of time that participants displayed positive or negative expressions and the total number of peaks per minute elicited, respectively.

Finally, explicit measurements were analysed by means of an ANOVA with Tukey’s test to find statistical differences among participants’ acceptability and purchase intention of the four packaging types, whereas a Kruskal–Wallis test with Dunn’s post hoc test was used for ranking data.

## 3. Results and Discussion

### 3.1. Respondents’ Characteristics

The sociodemographic characteristics of participants in both studies (packaging design and validation process) are shown in Table 1. The final sample of the first study matched the quota set for gender. The proportion of participants within each age category was similar to that of the Spanish’s population [66]. The sample was biased towards higher-educated individuals (57.0%), a percentage that largely exceeded the average of Spain (35.1%). As stated by Claret et al. [67], this bias may be caused by the higher self-confidence and willingness to participate in consumer studies as education level increases, a trend that has also been found in other studies [68]. The sample was also biased towards households with 3–4 people (64.0%), a higher proportion than the national average (38.1%). Participants with children at home (55.5%) exceeded the national average (45.4%), a fact that may be explained by the larger household size.

On the other hand, in the validation study, women were overrepresented, and the proportion of participants within each age category differed from the Spanish one [66]. Nevertheless, the representativeness of the population was not pursued, as the sample size was relatively small (*N* = 40), although it was large enough to find significant differences [69]. The number of participants in implicit measurement experiments is usually low due to the time required to individually gather the data from each participant, normally ranging from 30 to 75 respondents [34,70].

### 3.2. Visual Attributes

The visual attributes of the packaging most widely selected by respondents are presented in Table 2. The preferred container for the fish burger was the tray, chosen 66.5% of times, followed by the box (20.5%) and the bowl (7.0%). The participants’ habits may have played a relevant role in their decision, as the tray is also the most common container for fresh burgers in Spanish supermarkets [71].

Colour white (RGB: R (255), G (255), B (255)) and light blue (R (0), G (153), B (255)) had a similar preference, as they were selected on a comparable number of occasions (12.5% and 10.0%, respectively), followed by dark blue (R (0), G (0), B (255)), chosen 6.0% of times. According to Heide and Olsen [11], blue is widely used on fish packaging as it may be related with the ocean and water. It is highly common for people to associate specific colours to certain product categories or characteristics such as simplicity, cleanness and hygiene and white colour [72].

Participants showed a preference for seeing the raw burger through a full window on the package (29.5%) but also to see it through a large left side window (12.0%) or a circular central one (10.0%). The results obtained were similar to those reported by Arvanitoyannis et al. [47], where 77% of Greek consumers preferred transparent packaging for fish. Globally, approximately 55% of food products launched on the market have a window or transparent packaging to observe the product [73], a trend that is set to continue.

Food packaging enables consumers to see the product not only through a window on the package but also through pictures printed on the label [7]. Most participants chose to observe a higher number of times pictures of ready-to-eat dishes (48.0%) and raw ingredients of the burgers (35.5%) depicted on the package. One of the advantages of using a picture of raw ingredients is that consumers can easily identify them, allowing for the differentiation from other processed products with similar formats (for example, meat burgers).

Typeface is a ubiquitous element of packaging design that may impact consumers’ perceptions and that plays a key role in setting the visual identity of brands and companies (for example, logo) [14,15]. In the present study, the typefaces most selected were “Arial Rounded” (17.0%), closely followed by “Rage Italic” (11.5%) and “Edwardian Script ITC” (10.5%). Broadly speaking, consumers preferred two classes of typefaces: upright (for example, Arial Rounded) and script/handwriting style (for example, Rage Italic and Edwardian Script ITC). Script are casual typefaces that are not suited for long-written documents, as neither are italics, mainly because we are not used to it [74]. As stated by the same author, “we read best what we read most”. Consequently, although participants from the present study showed their preference for style script typefaces, the results should be used with caution, being advisable to only use script typefaces and italics for short statements but not for long sentences on the packaging.

The preferred products’ presentation was packaged per serving (two burgers per serving) (43.0%), closely followed by individually packaged (36.5%) and without divisions (20.5%), that is, all burgers together inside the package. 

Finally, the quantities of product picked out a higher number of times were two (45%) and four servings (38.0%). Those results may be explained as 75.5% of participants having three or more people living in their household and, therefore, requiring a greater quantity of the product. Silayoi and Speece [8] already found that small households tend to choose smaller packages, as they related larger food proportions with waste. The importance of the quantity of the product also lies in its influence on consumption, as the packaging size determines the frequency of purchasing [75].

### 3.3. Textual Attributes

The textual attributes of the packaging found to be more important for participants are presented in Table 3.

#### 3.3.1. Searched Quality

“Recyclability” (29.36%) and “price” factors (29.31%) played the most relevant roles in terms of searched quality, as they presented the highest relative importance. According to Luttenberger [22], 69% of food and drink launched on the global market in 2020 included some claim about the environment, a fact that may have caused consumers to consider it irrelevant or, at least, overlooked in the packaging. However, far from being overlooked, participants in the present study placed the highest relative importance for the “recyclability” factor. 

As already reported by Conte et al. [76], price has been found to be among the most relevant criteria for consumers when buying any type of goods, including fish. In addition, convenience was also pointed out as one of the main reasons for purchasing fish products [76]. Even so, in the present study the “convenience” factor showed a low relative importance (18.87%). This result contrasts with various studies which state that many consumers perceive difficulties in the preparation of fish, especially those with a low degree of self-efficacy (that is, how capable an individual feels when dealing with a specific situation) [46]. Therefore, it was expected that the “convenience” factor would have greater importance for fish-based products. However, fish burger format is widely available and well-established in Spanish markets, consequently consumers should be aware of its convenience. 

#### 3.3.2. Experienced Quality

Fish freshness is regarded as a key criterion of fish quality [77], mainly due to the fact of its high perishability [67]. Moreover, findings from the present study showed that “freshness” was the most important factor for experienced quality (29.82%). 

The “novelty” factor also played a relevant role (27.39%). These results may be explained by the fact that the “meagre burger with black trumpet” is a novel product in Spanish markets, mainly for two reasons: (i) meagre is a fish species little-known and produced in Spain [78]; and (ii) black trumpet is an unusual type of mushroom. 

It is widely known that texture of fresh fish is crucial for consumers’ acceptance [79], although there is more uncertainty when it comes to the texture of processed products. Results from the present study showed that “texture” was relegated as one of the less important factors (19.27%) when compared with the rest of the experienced quality factors.

#### 3.3.3. Credential Quality

“Animal welfare” (31.05%) and “health” factors (25.14%) stood out for credential quality. Animal welfare has been gaining attention over the last decades and, currently, plays a relevant role in consumers’ food choices [80]. Stubbe and Yang [81] found that consumers would increase their consumption if they knew fish had been raised ensuring animal welfare, and were even willing to pay 25% extra. In contrast, health has for a long time been a main driver of the consumption of fish [82]. According to Morrison, Bjerkas and Maddan [83], health may be one of the main competitive advantages of fish over other food goods. In Europe, the belief that eating fish is healthy remains widespread. Indeed, a cross-cultural study carried out by Pieniak, Verbeke and Scholderer [84] brought to light that Spanish consumers were among the most interested in healthy eating. 

Surprisingly, the “sustainability” factor had low relative importance (23.09%), contrary to what was expected. Previous literature revealed that, although sustainability was a relevant attribute for consumers, it did not shape their consumption of fish [85]. According to Luttenberger [22], the term “sustainable” and its derivates have been misused and overused, thus causing consumers’ numbness and indifference to the whole concept of sustainability. Evidence for this fact is that the respondents from this study placed a high importance on recyclability, which belongs to the environmental sustainability pillar [21], but at the same time, they placed little importance to the overall concept of sustainability.

#### 3.3.4. Claims: Informative vs. Interpretative

The results showed that 5 of the 12 factors presented significant differences between levels (price, recyclability, novelty, animal welfare and sustainability), the informative claims being always preferred over the interpretative ones (Table 4). The results obtained differed from those of Egnell et al. [20] and Feunekes et al. [18], who found that front-of-package interpretative labels outperformed informative ones. However, it should be noted that these studies were focused on nutritional information, which may move away from the aim of the present study, even though they are worth mentioning. When it comes to nutritional information, nutrient-specific labels (informative) that only provide numerical information are often poorly understood by consumers [20]. However, in the present study, it is unlikely that consumers had difficulties in interpreting the informative claims because most of them did not contain numerical values. Only one claim contained nutritional values (over 0.6 g of Omega-3 fatty acids), although this informative claim did not differ in consumer preferences from the interpretative claim “Protects your heart”.

Cultural aspects linked to different regions or countries also may have played a relevant role in the preferences of Spanish consumers for informative claims. As early research has already shown, there is an influence of cultural aspects on the perception of claims and labels on food products [18,20,86,87].

### 3.4. Validation of Co-Created Packaging Design

As previously mentioned, four packaging mock-ups were used as an input for the validation process. Those were labelled in short as: (i) white tray: packaging of the preferred visual and textual attributes chosen by participants; (ii) blue carton: packaging of the second preferred visual and textual attributes chosen by participants; (iii) black tray: photo edited packaging from an owned brand on the Spanish market; (iv) bag: photo edited packaging from the best-known brand of fish products in Spain.

#### 3.4.1. Visual Attention: Simultaneous Packaging Presentation

**Figure 2:**
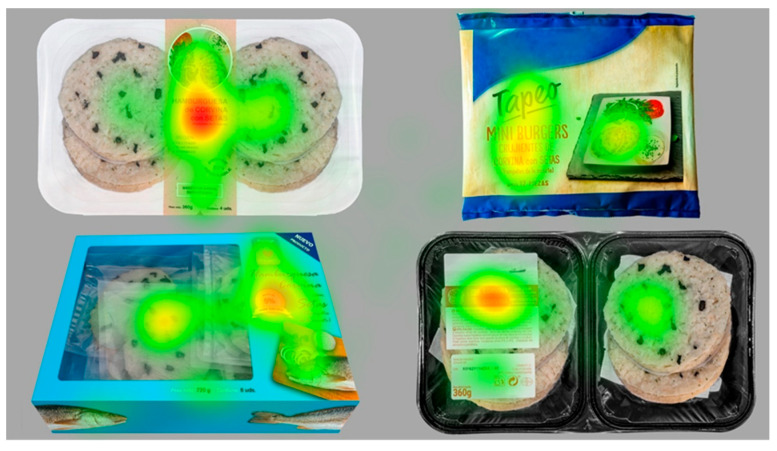
Heatmap (visual attention) for the four packaging images presented simultaneously. Visual attention ranged from low (green colour) to high (red colour). Text is in Spanish.

The simultaneous presentation of the packages tried to emulate a real situation in a supermarket shelf in which consumers deal with multiple products at a time. The heatmap showed the visual attention paid to the four packaging images when presented simultaneously: the redder the area, the higher the visual attention (Figure 2). Qualitatively, the packaging that caught the most of the participants’ attention was the white tray, although a more objective approach was provided by quantitative eye tracking metrics (Table 5). The shorter the TTFF, the faster the packaging caught the participant’s attention.

In the present study, both packages design co-created by participants (white tray and blue carton) caught the participants’ attention first and were revisited more frequently, although no significant differences were found between the four packaging at the fixed *p*-value < 0.050. Gigerenzer [88] advised that the significance level should not be fixed by convention at 0.050 in any discipline but not especially in social science, as always depend on the aim of the research and the sample size. Although 0.050 is the most widespread value to set the significance level, other authors opt for considering using higher *p*-values, such as 0.100 or 0.200 [89,90,91]. It should be noted that the statistical probability values obtained for the non-significant variables in our case were 0.299 for revisit count and 0.220 for the TTFF, indicating those values certain discriminant ability. In addition, it should be kept in mind that the number of participants in implicit measurement experiments is usually low (*N* = 40 in this study) and that the measures tend to show a higher inter-individual variability. Finally, the fixation count was higher for the blue carton (*p* < 0.0001), meaning that it elicited higher amounts of attention. Broadly speaking, the results obtained indicated that there were no major differences between the two co-created packages and the two most well-established existing fish products in the Spanish market in terms of capturing the participants’ visual attention. Therefore, considering that capturing consumers’ attention is the first step in arousing interest in a product [92], the co-created products have similar likelihood of being considered during the buying decision process.

#### 3.4.2. Visual Attention: Individual Packaging Presentation

**Figure 3:**
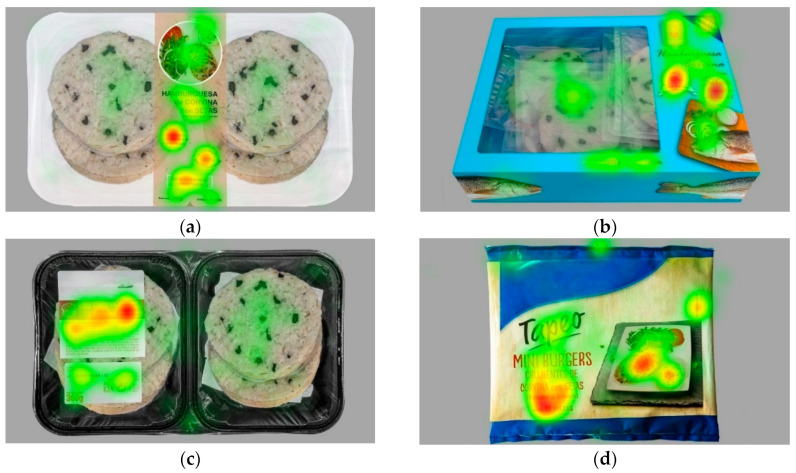
Individual heatmap (visual attention): (**a**) white tray; (**b**) blue carton; (**c**) black tray; (**d**) bag. Visual attention ranged from low (green colour) to high (red colour).

When individual packaging images were presented, the larger size of the pictures displayed a greater level of detail. The individual heatmap of the four packages allowed for the discovery that for the packages co-created by consumers (white tray and blue carton), the textual attributes drew a lot of attention (such as freshly filleted, processed and packed; 80% recyclable packaging; guaranteed animal welfare; now 5% cheaper) (Figure 3). In the same vein, visual cues (such as quantity, presentation, picture, and window) also grabbed participants’ attention. Nevertheless, some visual cues were difficult to evaluate with ET, for example, it was not possible to determine if participants were looking at the type of container or its colour. For the mock-ups of the competitors, the name of the mushroom “black trumpet”, which in Spanish means “trumpets of death”, obtained the most attention together with the ready-to-eat picture, serving suggestion text, fish species and instructions for use.

Nevertheless, although attention by individuals is a gateway to a higher-order cognitive process of the information [92], seeing is not necessarily liking [93]. Therefore, complementing ET measurements with other implicit (for example, emotional response) and explicit techniques allowed to more accurately capturing consumers’ opinions and preferences.

#### 3.4.3. Emotional Response: Intensity and Valence

**Figure 4:**
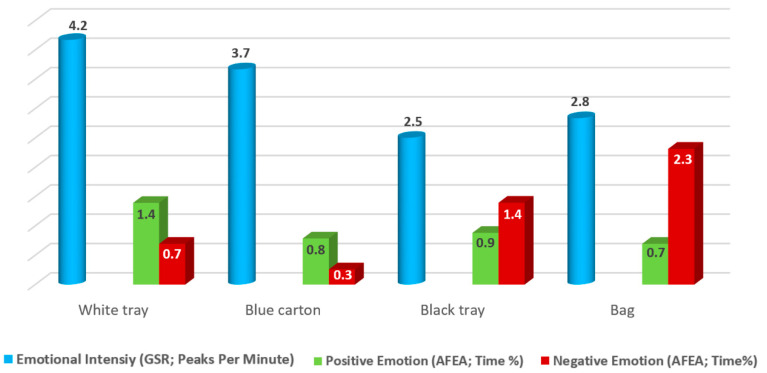
Intensity and valence (positive and negative) of the emotions elicited by the packaging (individual presentation).

Emotional intensity indicated how many emotional activation events occurred every minute. The higher the number, the more intense the participant’s emotional response. The valence of the emotional response was expressed as the percentage of time that participants displayed a positive or negative facial expression. Results showed that no statistically significant differences were found in GSR (*p* = 0.246) and AFEA (*p* = 0.481) measurements among the four packaging (Figure 4). Two main reasons may explain the results obtained, (i) all packages elicited a similar emotional intensity and valence; (ii) the type of stimuli used (images) were not engaging-enough to arise intense emotional responses. Some images are able to arise emotional responses, especially disgusting or shocking ones, whereas other images require more contextual information [43].

#### 3.4.4. Explicit Measurements

To infer which attributes from the packaging conveyed positive and negative emotions from the participants, the results from individual interviews were used, connecting implicit and explicit results; the most recurrent ones are listed in Table 6. The results showed that all positive aspects elicited for the two packages co-created by participants were visual (such as ready-to-eat picture, individually packed and fish picture) or textual attributes (such as “80% recyclable packaging”, “guaranteed animal welfare” and “protects your heart”). Only “now 5% cheaper” conveyed negative feelings among participants. Most of them argued that they were distrustful, one participant stated, “do they save on the quality of the product as well?”, while others hesitated if claim should not be permanently printed on the label.

The results of the self-reported explicit measurements (Table 5) showed a clear preference of participants for one of the packaging co-created by consumers in the former experiment. In particular, the preferred option was the white tray, which obtained the best scores in the three explicit measurements: ranking, acceptability, and purchase intention.

### 3.5. Limitations and Suggestions for Future Research

This study is exploratory in nature and not without limitations. The recruitment of the 200 participants was made by means of a convenience sampling, therefore, generalisation of the results to the studied population should be made with caution. Because of the relatively low number of consumers recruited, it has not been possible to carry out a deeper analysis to identify consumer segments with differentiated preferences and design different packaging tailored for specific consumers’ needs.

The inclusion of multiple attributes in the co-created packages allowed us to design a realistic packaging and to test it against real competitor products in the market, although it limits the possibility to assess interactions between attributes or to identify which combination of them may play the most important role in shaping participants’ preferences.

In the questionnaire of the former study, participants were only offered the Spanish word “pescado” (fish in English) to select the typeface they preferred. Results obtained showed that participants frequently chose style script typefaces, which are not suited for long-written documents due to their poor readability. Thus, results might have changed if they had been presented with a full statement that also allowed them to assess the legibility of the typeface. Introducing this change in the questionnaire is highly advisable for future studies.

Finally, the ecological validity of data gathered through surveys or in artificial laboratory setting can be easily argued. In this sense, sometimes the stimuli used in the implicit measurements (for example, fish product pictures), may be not engaging enough to arise a measurable emotional response. Therefore, future studies should focus on more realistic settings, such as a real shopping environment, thus including physical prototypes to better confirm or reject the usefulness of incorporating consumers through co-creation in the design of the packages.

## 4. Conclusions

A co-creation process with consumers was successfully applied for packaging design, which allowed for the independent identification of the visual and textual attributes that best fit their preferences. Participants had clear preferences for specific package attributes for a fish product, both in terms of visual and textual attributes. The information gathered could be useful for designers and marketers to guide the packaging design for fish products in the Spanish market, although care is required if the results obtained are extrapolated to other countries and products, as there may be significant differences among consumers’ preferences from different regions and cultures. Consumers showed their preference for informative claims over interpretative ones when it comes to the packaging of a fish product.

The validation process carried out with implicit and explicit methods showed that the packaging co-created by consumers were equally or even more preferred than two of the competitor fish products from the Spanish market. The use of implicit techniques allowed us to obtain measurements without the interference of social conventions. The results obtained indicated that co-created packaging captured participants’ visual attention and emotional response in a similar way to that of the competitor fish products. Explicit measurements showed that the co-created packaging designed according to the participants’ selected visual and textual attributes (white tray) was the mock-up preferred in the validation study, showing a higher acceptability and purchasing intention. Therefore, the validation performed confirmed the usefulness of incorporating consumers’ opinions during packaging design.

As a final conclusion, it is highly encouraged to involve consumers in all stages of the NPD, but especially in packaging design, as it has been proven that co-creation is a straightforward and effective way to design the fish product’s packaging according to consumers’ needs and demands, a fact that could improve a products’ success on the market.

## Figures and Tables

**Table 1:** Sociodemographic characteristics of the participants in studies 1 (packaging design) and 2 (validation process) expressed as a percentage.

Sociodemographic Characteristics (%)		Study 1 (*N* = 200)	Study 2 (*N* = 40)
Gender	Men	50.0	37.5
	Women	50.0	62.5
Age	18–24	11.0	2.5
	25–34	25.5	37.5
	35–44	22.5	22.5
	45–54	21.0	15.0
	55–64	20.0	22.5
Education level	Non-university	43.0	50.0
	University or higher	57.0	50.0
Household size	1–2	24.5	50.0
	3–4	64.0	40.0
	>4	11.5	10.0
Children at home	Yes	55.5	30.0
	No	44.5	70.0

**Table 2:** Percentage of the three most frequently chosen options within each visual attribute of the packaging.

Attribute	%	Option
Container	66.5	Tray
	20.5	Box
	7.0	Bowl
Colour	12.5	White
	10.0	Light blue
	6.0	Dark blue
Window	29.5	Full window
	12.0	Large left side window
	10.0	Circular central
Picture	48.0	Dish ready-to-eat
	35.5	Ingredients
	8.0	Other pictures
Typeface	17.0	Arial Rounded MT Bold
	11.5	Rage Italic
	10.5	Edwardian Script ITC
Presentation	43.0	Packed per serving
	36.5	Individually packed
	20.5	Without divisions
Quantity	45.0	2 servings
	38.0	4 servings
	9.0	1 serving

**Table 3:** Relative importance (RI) (%) of the factors from the textual analysis grouped by quality dimension.

Searched Quality		Experienced Quality		Credential Quality	
Factor	RI (%)	Factor	RI (%)	Factor	RI (%)
Convenience	18.87 ^b^	Freshness	29.82 ^a^	Health	25.14 ^a,b^
Price	29.31 ^a^	Texture	19.27 ^c^	Natural	20.72 ^b^
Presentation	22.46 ^b^	Flavour	23.52 ^b,c^	Animal welfare	31.05 ^a^
Recyclability	29.36 ^a^	Novelty	27.39 ^a,b^	Sustainability	23.09 ^b^

^a–c^ Different letters in the same column indicate statistically significant differences (*p* < 0.050).

**Table 4:** Mean utility of the levels of the factors from the textual analysis grouped by quality dimension.

Quality	Factor	Information Type	Level	Utility Mean
Searched	Convenience	Informative	Ready in 5 min	−0.09
		Interpretative	Quick to prepare	0.09
	Price	Informative	Now 5% cheaper	0.23 ^a^
		Interpretative	Your wallet will appreciate it	−0.23 ^b^
	Presentation	Informative	Contains 4 individually wrapped portions	0.05
		Interpretative	Packaging adapted to your pace of life	−0.05
	Recyclability	Informative	80% recyclable packaging	0.36 ^a^
		Interpretative	For a world with less plastic	−0.36 ^b^
Experienced	Freshness	Informative	Freshly filleted, processed and packed	0.11
		Interpretative	Unique freshness	−0.11
	Texture	Informative	Juicy	−0.01
		Interpretative	Incredibly juicy	0.01
	Flavour	Informative	With all the fish flavour	0.10
		Interpretative	Delicious	−0.10
	Novelty	Informative	New product	0.15 ^a^
		Interpretative	Enjoy something new	−0.15 ^b^
Credential	Health	Informative	Over 0.6 g of Omega-3 fatty acids	−0.01
		Interpretative	Protects your heart	0.01
	Natural	Informative	No colourant or preservatives	−0.02
		Interpretative	Only natural ingredients	0.02
	Animal welfare	Informative	Guaranteed animal welfare	0.38 ^a^
		Interpretative	Our fish are happy and you can tell	−0.38 ^b^
	Sustainability	Informative	Sustainable fish	0.17 ^a^
		Interpretative	Fish for today and tomorrow	−0.17 ^b^

^a,b^ Different letters in the same column within the same factor indicate that the informative and interpretative levels were significantly different (*p* < 0.050); the absence of letters indicate no statistical differences.

**Table 5:** Implicit measurements (eye tracking metrics) and explicit measurements for the four packaging images presented simultaneously.

Packaging	Implicit Measurements			Explicit Measurements			
	TTFF (ms)	Fixation Count	Revisit Count	Ranking	Mean Score Ranking	Acceptability	Purchase Intention
White tray	6955.04	723 ^b^	161	1	1.88 ^a^	6.55 ^a^	6.65 ^a^
Blue carton	7073.16	937 ^a^	164	2	2.45 ^a,b^	5.85 ^a,b^	5.00 ^b^
Black tray	7710.72	757 ^b^	146	3	2.75 ^b^	5.38 ^b^	5.25 ^b^
Bag	7755.21	755 ^b^	135	4	2.93 ^b^	4.71 ^b^	5.13 ^b^

^a^^,b^ Different letters in the same column indicate statistically significant differences (*p* < 0.050); acceptability ranged from 0 (lowest acceptability) to 10 (maximum acceptability). Purchase intention ranged from 0 (absolutely no chance) to 10 (absolute certain to buy).

**Table 6:** Most common positive and negative opinions about the packaging gathered through individual interviews.

Packaging	Positive Opinion	Negative Opinion
White tray	“80% recyclable packaging” claim “Guaranteed animal welfare” claim Ready-to-eat picture	Valuable information was missing Uncertain about packing per serving or no division
Blue carton	Individually packed “Protects your heart” claim Fish picture	Too much plastic Mistrust due to the “now 5% cheaper” claim Valuable information was missing
Black tray	Packed per serving Lot of information at first sight Full vision of the product	Black colour of the tray Small letter size Too much plastic
Bag	Ready-to-eat picture “Tapeo” as the product’s description Blue colour	Unable to see the product Bag format container Information was missing

**Figure A1:**
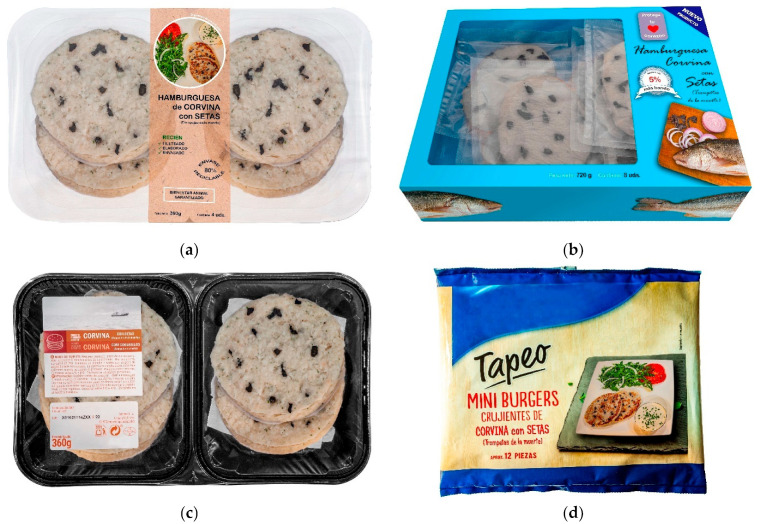
Mock-ups used as stimuli in the validation process: (**a**) packaging of the preferred visual and textual attributes chosen by participants; (**b**) packaging of the second preferred visual and textual attributes chosen by participants; (**c**) photo edited packaging from an owned brand in Spanish supermarkets with the highest sales share; (**d**) photo edited packaging of the best-known and well-established brand of fish products in Spain.

## Data Availability

Data is contained within the article.

## Appendix A

Questionnaire to assess participants’ preferences for visual attributes for a fish burger packaging. The full questionnaire can be obtained from the first author of this paper upon request.

Imagine you could design the appearance of your favourite packaging. For a sea and mountain burger: fish (meagre) with mushrooms (black trumpets), choose your favourite option from those presented in each question.

(1) Container type

For a sea and mountain burger: fish (meagre) with mushrooms (black trumpets), which container would you favour the most? Please select your preferred option from those presented below.

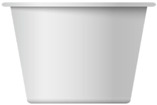
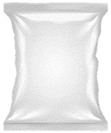
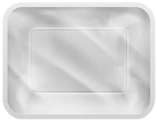
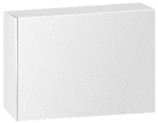

			
□ Bowl	□ Bag	□ Tray	□ Box

(2) Container colour

For a sea and mountain burger: fish (meagre) with mushrooms (black trumpets), which colour of the container would you favour the most? Please select your preferred option from those presented below.

(3) Window presence and type

For a sea and mountain burger: fish (meagre) with mushrooms (black trumpets), choose whether you would prefer the packaging to have a window and, if so, which type of window? Please select your preferred option from those presented below.

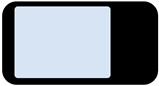
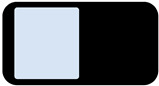
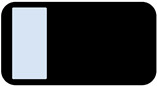
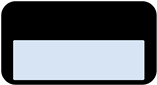

			
1. Large left side window	2. Medium left side window	3. Small left side window	4. Lower medium window

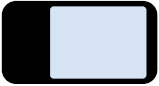
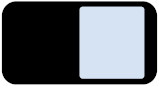
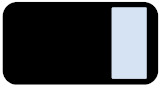
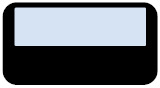
			
5. Large right side window	6. Medium right side window	7. Small right side window	8. Upper medium window

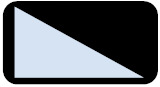
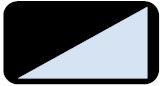
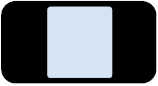
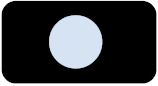
			
9. Triangular left side window	10. Triangular right side window	11. Central medium window	12. Circular central

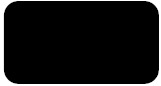
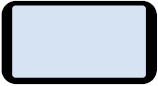
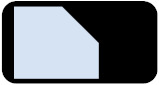
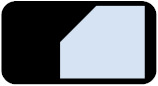
			
13. No window	14. Full window	15. Asymmetrical left side window	16. Asymmetrical right side window

(4) Picture presence and type

For a sea and mountain burger: fish (meagre) with mushrooms (black trumpets), choose whether you would prefer the packaging to have a picture and, if so, which type of picture? Please select your preferred option from those presented below.

1. Dish ready-to-eat;
2. Ingredients (e.g., fish, black trumpet…);
3. People (e.g., someone eating, a family…);
4. Other pictures (e.g., a landscape, the sea…);
5. A combination of the latter options;
6. Without a picture.

(5) Typeface

For a sea and mountain burger: fish (meagre) with mushrooms (black trumpets), which typeface would you favour the most? Please select your preferred option from those presented below.

1.		2.		3.	
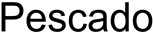
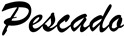
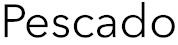
4.		5.		6.	
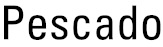
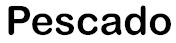
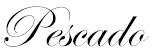
7.		8.		9.	
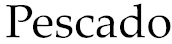
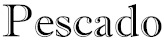
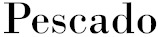
10.		11.		12.	
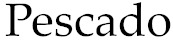
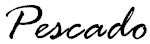
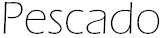
13.		14.		15.	
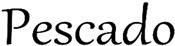
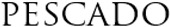
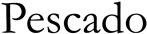

(6) Package presentation

For a sea and mountain burger: fish (meagre) with mushrooms (black trumpets), which type of package presentation would you favour the most? Please select your preferred option from those presented below.

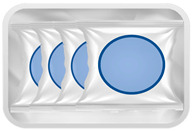
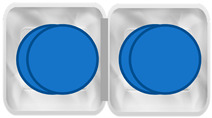
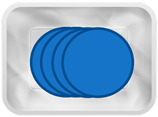

		
1. Individually packed	2. Packed per serving (two burgers per serving)	3. Without divisions, all burgers in the package together

(7) Quantity of the package

For a sea and mountain burger: fish (meagre) with mushrooms (black trumpets), which number of servings would you prefer? Consider two burgers per serving. Please select your preferred option from those presented below.

☐ 1 serving
☐ 2 servings
☐ 4 servings
☐ More than 4 servings

## Appendix B

The stimuli in full resolution can be obtained from the first author of this paper upon request.

## References

[1] POPAI (2014). Mass Merchant Shopper Engagement Study.

[2] Hallez L., Qutteina Y., Raedschelders M., Boen F., Smits T. (2020). That’s my cue to eat: A systematic review of the persuasiveness of front-of-pack cues on food packages for children vs. adults. Nutrients.

[3] van Rompay T.J.L., Veltkamp M. (2014). Product packaging metaphors: Effects of ambiguity and explanatory information on consumer appreciation and brand perception. Psychol. Mark..

[4] Ares G., Deliza R. (2010). Studying the influence of package shape and colour on consumer expectations of milk desserts using word association and conjoint analysis. Food Qual. Prefer..

[5] Butkevičiene V., Stravinskiene J., Rutelione A. (2008). Impact of consumer package communication on consumer decision making process. Eng. Econ..

[6] Newman C.L., Howlett E., Burton S. (2016). Effects of objective and evaluative front-of-package cues on food evaluation and choice: The moderating influence of comparative and noncomparative processing contexts. J. Consum. Res..

[7] Simmonds G., Spence C. (2017). Thinking inside the box: How seeing products on, or through, the packaging influences consumer perceptions and purchase behaviour. Food Qual. Prefer..

[8] Silayoi P., Speece M. (2004). Packaging and purchase decisions: An exploratory study on the impact of involvement level and time pressure. Br. Food J..

[9] Labbe D., Pineau N., Martin N. (2013). Food expected naturalness: Impact of visual, tactile and auditory packaging material properties and role of perceptual interactions. Food Qual. Prefer..

[10] Kees J., Royne M.B., Cho Y.N. (2014). Regulating front-of-package nutrition information disclosures: A test of industry self-regulation vs. other popular options. J. Consum. Aff..

[11] Heide M., Olsen S.O. (2017). Influence of packaging attributes on consumer evaluation of fresh cod. Food Qual. Prefer..

[12] Chrysochou P., Grunert K.G. (2014). Health-related ad information and health motivation effects on product evaluations. J. Bus. Res..

[13] Fenko A., de Vries R., van Rompay T. (2018). How strong is your coffee? The influence of visual metaphors and textual claims on consumers’ flavor perception and product evaluation. Front. Psychol..

[14] Celhay F., Boysselle J., Cohen J. (2015). Food packages and communication through typeface design: The exoticism of exotypes. Food Qual. Prefer..

[15] Velasco C., Hyndman S., Spence C. (2018). The role of typeface curvilinearity on taste expectations and perception. Int. J. Gastron. Food Sci..

[16] Ilyuk V., Block L. (2016). The effects of single-serve packaging on consumption closure and judgments of product efficacy. J. Consum. Res..

[17] Grunert K.G., Scholderer J., Rogeaux M. (2011). Determinants of consumer understanding of health claims. Appetite.

[18] Feunekes G.I.J., Gortemaker I.A., Willems A.A., Lion R., van den Kommer M. (2008). Front-of-pack nutrition labelling: Testing effectiveness of different nutrition labelling formats front-of-pack in four European countries. Appetite.

[19] Rybak G., Burton S., Johnson A.M., Berry C. (2021). Promoted claims on food product packaging: Comparing direct and indirect effects of processing and nutrient content claims. J. Bus. Res..

[20] Egnell M., Talati Z., Hercberg S., Pettigrew S., Julia C. (2018). Objective understanding of front-of-package nutrition labels: An international comparative experimental study across 12 countries. Nutrients.

[21] Hanss D., Böhm G. (2012). Sustainability seen from the perspective of consumers. Int. J. Consum. Stud..

[22] Luttenberger D. (2021). 2021 Packaging Trend: The Rise of Responsibility.

[23] Nielsen (2018). Setting the Record Straight on Innovation Failure.

[24] Moon H., Johnson J.L., Mariadoss B.J., Cullen J.B. (2018). Supplier and customer involvement in New Product Development stages: Implications for new product innovation outcomes. Int. J. Innov. Technol. Manag..

[25] van Kleef E., van Trijp H.C.M., Luning P. (2005). Consumer research in the early stages of new product development: A critical review of methods and techniques. Food Qual. Prefer..

[26] Hoyer W.D., Chandy R., Dorotic M., Krafft M., Singh S.S. (2010). Consumer cocreation in new product development. J. Serv. Res..

[27] Witell L., Kristensson P., Gustafsson A., Löfgren M. (2011). Idea generation: Customer co-creation versus traditional market research techniques. J. Serv. Manag..

[28] Banović M., Krystallis A., Guerrero L., Reinders M.J. (2016). Consumers as co-creators of new product ideas: An application of projective and creative research techniques. Food Res. Int..

[29] de Wijk R.A., Noldus L.P.J.J. (2021). Using implicit rather than explicit measures of emotions. Food Qual. Prefer..

[30] Piqueras-Fiszman B., Velasco C., Salgado-Montejo A., Spence C. (2013). Using combined eye tracking and word association in order to assess novel packaging solutions: A case study involving jam jars. Food Qual. Prefer..

[31] Donoghue S. (2000). Projective techniques in consumer research. J. Fam. Ecol. Consum. Sci..

[32] Ares G., Giménez A., Bruzzone F., Vidal L., Antúnez L., Maiche A. (2013). Consumer visual processing of food labels: Results from an eye-tracking study. J. Sens. Stud..

[33] Liao L.X., Corsi A.M., Chrysochou P., Lockshin L. (2015). Emotional responses towards food packaging: A joint application of self-report and physiological measures of emotion. Food Qual. Prefer..

[34] Kessler S.J., Jiang F., Andrew Hurley R. (2020). The state of automated facial expression analysis (AFEA) in evaluating consumer packaged beverages. Beverages.

[35] Pentus K., Mehine T., Kuusik A. (2014). Considering emotions in product package design through combining conjoint analysis with psycho physiological measurements. Procedia-Soc. Behav. Sci..

[36] Vergura D.T., Luceri B. (2018). Product packaging and consumers’ emotional response. Does spatial representation influence product evaluation and choice?. J. Consum. Mark..

[37] Russo E.J., Kent Hunt A.A. (1978). Eye fixations can save the world: A critical evaluation and a comparison between eye fixations and other information processing methodologies. Advances in Consumer Research.

[38] Caruelle D., Gustafsson A., Shams P., Lervik-Olsen L. (2019). The use of electrodermal activity (EDA) measurement to understand consumer emotions—A literature review and a call for action. J. Bus. Res..

[39] Critchley H.D. (2002). Electrodermal responses: What happens in the brain. Neuroscientist.

[40] iMotions (2017). Facial Expression Analysis: The Complete Pocket Guide.

[41] Andrade C. (2018). Internal, external, and ecological validity in research design, conduct, and evaluation. Indian J. Psychol. Med..

[42] Spinelli S., Monteleone E., Ares G., Varela P. (2018). Emotional responses to products. Methods in Consumer Research, Volume 1: New Approaches to Classic Methods.

[43] Grühn D., Sharifian N., Meiselman H.L. (2016). Lists of emotional stimuli. Emotion Measurement.

[44] Marques da Rosa V., Spence C., Miletto Tonetto L. (2019). Influences of visual attributes of food packaging on consumer preference and associations with taste and healthiness. Int. J. Consum. Stud..

[45] Birch D., Lawley M., Hamblin D. (2012). Drivers and barriers to seafood consumption in Australia. J. Consum. Mark..

[46] Carlucci D., Nocella G., De Devitiis B., Viscecchia R., Bimbo F., Nardone G. (2015). Consumer purchasing behaviour towards fish and seafood products. Patterns and insights from a sample of international studies. Appetite.

[47] Arvanitoyannis I.S., Krystallis A., Panagiotaki P., Theodorou A.J. (2004). A marketing survey on Greek consumers’ attitudes towards fish. Aquac. Int..

[48] Duerrschmid K., Danner L., Ares G., Varela P. (2018). Eye tracking in consumer research. Methods in Consumer Research, Volume 2: Alternative Approaches and Special Applications.

[49] van Bommel R., Stieger M., Visalli M., de Wijk R., Jager G. (2020). Does the face show what the mind tells? A comparison between dynamic emotions obtained from facial expressions and Temporal Dominance of Emotions (TDE). Food Qual. Prefer..

[50] Jain A.K., Mahajan V., Malhotra N.K. (1979). Multiattribute preference models for consumer research: A synthesis. Adv. Consum. Res..

[51] Sattler H., Hensel-Börner S., Gustafsson A., Herrmann A., Huber F. (2000). A comparison of conjoint measurement with self-explicated approaches. Conjoint Measurement: Methods and Applications.

[52] Schlereth C., Eckert C., Schaaf R., Skiera B. (2014). Measurement of preferences with self-explicated approaches: A classification and merge of trade-off- and non-trade-off-based evaluation types. Eur. J. Oper. Res..

[53] Banks S. (1950). The relationships between preference and purchase of brands. Source J. Mark..

[54] Bernués A., Olaizola A., Corcoran K. (2003). Extrinsic attributes of red meat as indicators of quality in Europe: An application for market segmentation. Food Qual. Prefer..

[55] Fandos C., Flavián C. (2006). Intrinsic and extrinsic quality attributes, loyalty and buying intention: An analysis for a PDO product. Br. Food J..

[56] Fernqvist F., Ekelund L. (2014). Credence and the effect on consumer liking of food—A review. Food Qual. Prefer..

[57] Lee H.J., Yun Z.S. (2015). Consumers’ perceptions of organic food attributes and cognitive and affective attitudes as determinants of their purchase intentions toward organic food. Food Qual. Prefer..

[58] Juster F.T. (1966). Consumer Buying Intentions and Purchase Probability: An Experiment in Survey Design. J. Am. Stat. Assoc..

[59] European Parliament (2013). REGULATION (EU) No 1379/2013 of 11 December 2013 on the common organisation of the markets in fishery and aquaculture products, amending Council Regulations (EC) No 1184/2006 and (EC) No 1224/2009 and repealing Council Regulation (EC) No 104/2000. Off. J. Eur. Union.

[60] Macfie H.J., Bratchell N., Greenhoff K., Vallis L.V. (1989). Designs to balance the effect of order of presentation and first-order carry-over effects in hall tests. J. Sens. Stud..

[61] Vu T.M.H., Tu V.P., Duerrschmid K. (2016). Design factors influence consumers’ gazing behaviour and decision time in an eye-tracking test: A study on food images. Food Qual. Prefer..

[62] Farnsworth B. 10 Most Used Eye Tracking Metrics and Terms. https://imotions.com/blog/7-terms-metrics-eye-tracking/.

[63] Antúnez L., Vidal L., Sapolinski A., Giménez A., Maiche A., Ares G. (2013). How do design features influence consumer attention when looking for nutritional information on food labels? Results from an eye-tracking study on pan bread labels. Int. J. Food Sci. Nutr..

[64] Kulke L., Feyerabend D., Schacht A. (2020). A Comparison of the affectiva iMotions facial expression analysis software with EMG for identifying facial expressions of emotion. Front. Psychol..

[65] iMotions (2017). Galvanic Skin Response: The Complete Pocket Guide.

[66] EUROSTAT Population on 1 January by Age and Sex. https://appsso.eurostat.ec.europa.eu/nui/show.do?dataset=demo_pjan&lang=en.

[67] Claret A., Guerrero L., Aguirre E., Rincón L., Hernández M.D., Martínez I., Peleteiro J.B., Grau A., Rodríguez-Rodríguez C. (2012). Consumer preferences for sea fish using conjoint analysis: Exploratory study of the importance of country of origin, obtaining method, storage conditions and purchasing price. Food Qual. Prefer..

[68] Llauger M., Claret A., Bou R., López-Mas L., Guerrero L. (2021). Consumer attitudes toward consumption of meat products containing offal and offal extracts. Foods.

[69] Gacula M., Rutenbeck S. (2006). Sample size in consumer test and descriptive analysis. J. Sens. Stud..

[70] Gonzalez Viejo C., Fuentes S., Howell K., Torrico D.D., Dunshea F.R. (2019). Integration of non-invasive biometrics with sensory analysis techniques to assess acceptability of beer by consumers. Physiol. Behav..

[71] Mintel Global New Products Database (GNPD). https://www.gnpd.com/sinatra/search_results/?search_id=jGAUEhRTnx&page=0.

[72] Labrecque L.I., Milne G.R. (2012). Exciting red and competent blue: The importance of color in marketing. J. Acad. Mark. Sci..

[73] Luttenberger D. (2015). The Use of Windows on Packaging.

[74] Spiekermann E. (2014). Stop Stealing Sheep & Find Out How Type Works.

[75] Rao R., Wansink B., Jain S. (2012). Commentaries and rejoinder to “marketing of vice goods: A strategic analysis of the package size decision” by Sanjay Jain. Mark. Sci..

[76] Conte F., Passantino A., Longo S., Voslářová E. (2014). Consumers’ attitude towards fish meat. Ital. J. Food Saf..

[77] Lazo O. (2017). Development of New Products from Aquaculture Fish Species. Ph.D. Thesis.

[78] APROMAR (2020). La Acuicultura en España 2020.

[79] Saavedra M., Pereira T.G., Carvalho L.M., Pousão-Ferreira P., Grade A., Teixeira B., Quental-Ferreira H., Mendes R., Bandarra N., Gonçalves A. (2017). Wild and farmed meagre, *Argyrosomus regius*: A nutritional, sensory and histological assessment of quality differences. J. Food Compos. Anal..

[80] Altintzoglou T., Verbeke W., Vanhonacker F., Luten J. (2010). The image of fish from aquaculture among Europeans: Impact of exposure to balanced information. J. Aquat. Food Prod..

[81] Stubbe S., Yang Y. (2011). Consumers’ perception of farmed fish and willingness to pay for fish welfare. Br. Food J..

[82] Olsen S.O. (2003). Understanding the relationship between age and seafood consumption: The mediating role of attitude, health and involvement and convenience. Food Qual. Prefer..

[83] Morrison C., Bjerkas M., Maddan G., Luten J.B., Oehlenschläger J., Ólafsdóttir G. (2003). The view from some European multiple retailers and brand owners on quality and traceability of fish. Quality of Fish from Catch to Consumer: Labelling, Monitoring and Traceability.

[84] Pieniak Z., Verbeke W., Scholderer J. (2010). Health-related beliefs and consumer knowledge as determinants of fish consumption. J. Hum. Nutr. Diet..

[85] Verbeke W., Vanhonacker F., Sioen I., Van Camp J., De Henauw S. (2007). Perceived importance of sustainability and ethics related to fish: A consumer behavior perspective. Ambio.

[86] Grunert K.G., Fernández-Celemín L., Wills J.M., Bonsmann S.S.G., Nureeva L. (2010). Use and understanding of nutrition information on food labels in six European countries. J. Public Health.

[87] Grunert K.G., Hieke S., Wills J. (2014). Sustainability labels on food products: Consumer motivation, understanding and use. Food Policy.

[88] Gigerenzer G. (2004). Mindless statistics. J. Socio-Econ..

[89] Bi J. (2005). Similarity testing in sensory and consumer research. Food Qual. Prefer..

[90] Gustafson D.H., Hawkins R., Boberg E., Pingree S., Serlin R.E., Graziano F., Chan C.L. (1999). Impact of a patient-centered, computer-based health information/support system. Am. J. Prev. Med..

[91] Jamali M., Khan R. (2018). The impact of consumer interaction on social media on brand awareness and purchase intention! Case study of Samsung. J. Mark. Logist..

[92] Wedel M., Pieters R., Malhotra N.K. (2015). A review of eye-tracking research in marketing. Review of Marketing Research.

[93] Husić-Mehmedović M., Omeragić I., Batagelj Z., Kolar T. (2017). Seeing is not necessarily liking: Advancing research on package design with eye-tracking. J. Bus. Res..

